# NDM-1- and OXA-23-producing *Acinetobacter baumannii* in wastewater of a Nigerian hospital

**DOI:** 10.1128/spectrum.02381-23

**Published:** 2023-10-05

**Authors:** Erkison Ewomazino Odih, Gabriel Temitope Sunmonu, Iruka N. Okeke, Anders Dalsgaard

**Affiliations:** 1 Department of Veterinary and Animal Sciences, Faculty of Health and Medical Sciences, University of Copenhagen, Copenhagen, Denmark; 2 Global Health Research Unit for the Genomic Surveillance of Antimicrobial Resistance, Department of Pharmaceutical Microbiology, Faculty of Pharmacy, University of Ibadan, Ibadan, Oyo State, Nigeria; US Department of Agriculture, Athens, Georgia, USA

**Keywords:** carbapenem-resistant *Acinetobacter baumannii*, hospital wastewater, antimicrobial resistance, international clone, carbapenem resistance

## Abstract

Carbapenem-resistant *Acinetobacter baumannii* spp. are increasingly important pathogens with limited treatment options, and there is limited knowledge on the environmental factors contributing to their spread. We determined the occurrence of carbapenem-resistant *A. baumannii* in hospital wastewater and their phylogenetic relationships with clinical *A. baumannii* isolates. Grab samples of raw and treated hospital wastewater were collected monthly at the University College Hospital, Ibadan, Nigeria, between March 2021 and February 2022. *Acinetobacter baumannii* strains were selectively isolated and identified using VITEK2, and their whole genomes were sequenced on an Illumina platform. We performed antimicrobial susceptibility testing and *in silico* genomic characterization of the strains and determined their phylogenetic relationships to previously characterized clinical *A. baumannii* strains from Nigeria. *A. baumannii* complex isolates were recovered from wastewater throughout the study. Of the 82 isolates identified based on whole-genome sequences, 77 were *A. baumannii. A. baumannii* isolates had high resistance rates (≥48.1%) to 10 of 12 antimicrobials tested, and majority (42/77, 54.5%) were resistant to carbapenems, with *bla*_NDM-1_ being the most common (24/77, 31.2%) carbapenem resistance gene detected, followed by *bla*_OXA-23_ (*n* = 22, 28.6%). There was no statistically significant difference in carbapenem resistance rates or carbapenem gene carriage between the raw and treated wastewater isolates. Most of the isolates belonged to novel or sparsely described lineages, some of which were closely related to clinical isolates. The release of inadequately treated hospital wastewater into the environment may contribute to the increased spread of carbapenem-resistant and clinically important *A. baumannii* lineages in Ibadan, Nigeria.

**IMPORTANCE:**

*Acinetobacter baumannii* is a leading cause of hospital-associated infections globally. *A. baumannii* reservoirs outside hospital settings are still unknown, and their occurrence in the environment is linked to clinical and anthropogenic activities. Although the risk of transmission of *A. baumannii* from environmental sources to humans is not fully understood, these sources pose significant risks for the continued dissemination of *A. baumannii* and their resistance traits. This study provides evidence that diverse and clinically relevant *A. baumannii* strains, many of which are resistant to carbapenems, are constantly being discharged into the environment through inadequately treated hospital wastewater. We further elucidate potential transmission routes between the environment and clinical infections and demonstrate the high prevalence of carbapenem resistance genes on highly mobile transposons among these strains. Our findings highlight the pressing need to address hospital wastewater as a crucial factor in curtailing the spread of carbapenem-resistant *A. baumannii*.

## INTRODUCTION

*Acinetobacter baumannii* spp. are Gram-negative, notorious opportunistic pathogens that are increasingly implicated in drug-resistant hospital-acquired infections globally. They are highly adapted to harsh nutrient-deficient and desiccated environments and can thus survive for long periods on hospital surfaces (e.g., door handles, call buttons, and bed rails), medical devices, in biofilms in hospital taps, sinks, and drains, as well as on the skin or clothing of healthcare personnel or patients, from where they can easily be transmitted to people (1 – 6). In addition to their capacity to thrive and spread within hospital environments, the clinical significance of *A. baumannii* stems from their increasing global prevalence, high mortality rates, and frequent acquisition of antimicrobial resistance (AMR) determinants, including those conferring resistance to last-line antimicrobials such as carbapenems (7 – 9). These factors have led to the inclusion of carbapenem-resistant *A. baumannii* in the World Health Organization’s critical-priority list of pathogens for which new antimicrobials are urgently needed (10).

*Acinetobacter baumannii* therapy is typically problematic (11) and is further worsened by the increasing rates of resistance to carbapenems, with high resistance rates of up to 100% reported globally (12 – 15). Carbapenem resistance in *A. baumannii* is encoded primarily by *bla*_OXA-23_ genes, which are more common among the globally disseminated clones and are frequently mobilized and spread between different strains via plasmids (3, 8, 16). In 2019, a review of publicly available *A. baumannii* genomes showed that 82% of the 2,345 genomes with carbapenem resistance genes carried *bla*_OXA-23_ compared to lower proportions of other carbapenem resistance genes (8). Recent evidence has also revealed an increase in the prevalence of *bla*_NDM-1_ genes, which have a wider and more potent hydrolytic spectrum, among *A. baumannii* (14, 17, 18). Our previous study characterizing clinical *A. baumannii* isolates in the southwestern region of Nigeria showed a local *bla*_NDM-1_ prevalence of 27.9% compared to a 34.9% prevalence of *bla*_OXA-23_ (19). These high antimicrobial resistance rates and potential for continued spread is a significant public health concern as treatment options for infections caused by carbapenem-resistant *A. baumannii* are severely limited (11, 20, 21).

Knowledge of the environmental contributors to the spread of *A. baumannii* and the carbapenem resistance genes they frequently harbor is critical to guide the design and implementation of mitigation strategies. Multiple studies have demonstrated the presence of carbapenem-resistant and carbapenem-susceptible *A. baumannii* in wastewater sources globally, including hospital wastewater (22 – 26). In Nigeria, the presence of drug-resistant bacterial species, including *A. baumannii*, in multiple wastewater sources has also been reported (27, 28), but the importance of *A. baumannii* in hospital wastewater in Nigeria with respect to carbapenem resistance genes, lineage distribution, and their clonal relationships to hospital isolates remains unknown. The high persistence and adaptability of *A. baumannii* in low-nutrient environments such as water suggest that, if present in the final (un)treated wastewater released into the environment, these strains may persist, even multiply, and may be important sources of human exposure and subsequent infections (23, 29). Although the vast majority of *A. baumannii* infections are hospital-acquired, there is evidence of often fatal community acquisition and spread, especially in tropical regions (30 – 34). As such, the potential inadvertent and sustained release of carbapenem-resistant *A. baumannii* into the environment through hospital wastewater portends important public health ramifications. Establishment in these ecological niches, e.g., in biofilms, is associated with increased risk of spread of resistance genes on mobile genetic elements to other *A. baumannii* and non-*Acinetobacter* species (22, 35).

In addition to providing information on the potential risk of spread of carbapenem-resistant *A. baumannii* through wastewater, the detection of clinically important lineages of *A. baumannii* in wastewater may also provide a useful indicator of pathogens circulating in the human population (36, 37). This study thus aimed to determine the occurrence of carbapenem-resistant *A. baumannii* in hospital wastewater over 1 year, their characteristics and phylogenetic relationships to clinical *A. baumannii* isolates from the same region in Nigeria.

## MATERIALS AND METHODS

This study was conducted at the University College Hospital (UCH), Ibadan, Oyo State, Nigeria. The University College Hospital, Ibadan, is a tertiary hospital with an 1229-bed capacity (38). The wastewater treatment plant at the Environmental Health Department, UCH, processes 28,000 L of wastewater per day and employs a multi-step wastewater treatment process. Gross solids are first removed at the preliminary treatment step using a skimming tank, after which the remaining solids are removed in the primary treatment step using a combination of sedimentation, mechanical flocculation, and chemical coagulation methods. This is followed by aerobic oxidation treatment with activated sludge and anaerobic digestion. The digested sludge is separated and stabilized, and the wastewater is held in oxidation ponds and chlorinated before disposal. The final treated wastewater is discharged into the downstream Dandaru Reservoir, which is interconnected with a vast river network.

### Collection of hospital wastewater and isolation of *Acinetobacter baumannii*

Monthly grab samples (i.e., single, discrete samples collected at a particular time) of untreated and treated hospital wastewater were collected between (March 2021) and February 2022 from the wastewater treatment plant at UCH. Untreated (raw) wastewater samples were collected from the inlet flow point into the wastewater treatment plant, while treated samples were collected from the final treated wastewater effluent at the point of discharge. Raw and treated wastewater samples were collected on the same day each month in sterile 1-L wide-neck plastic containers and transported on ice to the laboratory for processing typically within 1 hour of collection. All samples were collected between 9 a.m. and 12 noon.

Ten-fold serial dilutions were prepared from the water samples and plated onto freshly prepared CHROMagar Acinetobacter media with CHROMagar MDR Supplement CR102 (CHROMagar, Paris, France) additionally supplemented with 2 µg/mL of cefotaxime to increase the chances of recovering cephalosporin-resistant *A. baumannii*. Plates were incubated at 37°C for 24 hours, after which all presumptive *A. baumannii* colonies (up to a maximum of 20 distinct colonies for each sample) were selected based on their colony morphology (red colonies) and sub-cultured onto antibiotic-free CHROMAgar plates and incubated at 37°C for 24 hours to obtain pure cultures. Pure cultures were cryopreserved at −80°C prior to further analyses.

### Identification of bacteria from wastewater

Presumptive *A. baumannii* isolates were identified using the GN ID (reference number: 21341) cards on the VITEK two automated system (bioMérieux, Inc., Marcy-l’Étoile, France) following the manufacturer’s instructions. Isolates identified as *Acinetobacter baumannii* complex were stored for downstream characterization.

### Antimicrobial susceptibility testing

The susceptibility of the *A. baumannii* complex isolates to selected antimicrobials was determined using the VITEK two automated system with the antimicrobial susceptibility testing (AST) N281 (reference number: 414531) cards according to the manufacturer’s instructions. The antimicrobials tested included cefepime, ceftazidime, ciprofloxacin, doripenem, gentamicin, imipenem, levofloxacin, meropenem, minocycline, piperacillin/tazobactam, ticarcillin/clavulanic acid, and tigecycline. Minimum inhibitory concentration (MIC) values were interpreted using the AMR R package version 1.8.1 (https://msberends.github.io/AMR/) according to the clinical breakpoints of the Clinical Laboratory Standards Institute (CLSI) (39), except for the tigecycline MIC values as the current guidelines by CLSI and the European Committee on Antimicrobial Susceptibility Testing (40) do not contain breakpoints for interpreting tigecycline MIC values for *A. baumannii*. Tigecycline MICs of 2 µg/mL were reported as resistant (41). All non-susceptible isolates are reported as resistant in the analyses.

### Whole-genome sequencing

Overnight cultures of *A. baumannii* complex isolates in tryptone soy vroth (Oxoid, Basingstoke, United Kingdom) were centrifuged at 6,000 revolutions per minute for 5 min, and the pellets were harvested for use as starting material for the DNA extraction using the FastDNA Spin Kit for Soil (MP Biomedicals, Irvine, CA, United States) according to the manufacturer’s instructions. Whole-genome sequencing libraries were prepared from the extracted DNA using the NEBNext Ultra II FS DNA library kit for Illumina (New England Biolabs, Ipswich, MA, United States) according to the manufacturer’s instructions, and the genomic libraries were sequenced on an Illumina MiSeq platform with 150-bp paired-end chemistry (Illumina, San Diego, CA, United States).

### Clinical *A. baumannii* isolates

We sought to assess the clinical significance of the wastewater isolates by determining their phylogenetic relationships to previously characterized clinical isolates in Nigeria. To do this, we retrieved the whole-genome sequences of 89 clinical *A. baumannii* isolates obtained from hospitals or laboratories across southwestern Nigeria between 2016 and 2022 . These included the genomes of 86 *A*. *baumannii* isolates characterized in our previous study (19) and 3 other additional isolates obtained from UCH, Ibadan. In total, 21 of the clinical isolates (including 18 previously characterized isolates) were from the UCH health facility, where the wastewater samples were collected, while the remaining 68 were from other healthcare facilities in different states within the same southwestern region of Nigeria. These facilities were Lagos University Teaching Hospital, Idi-Araba, Lagos State; Clina-Lancet Laboratories, Victoria Island, Lagos State; EL-LAB Medical Diagnostics, Festac, Lagos State; Obafemi Awolowo University Teaching Hospitals Complex, Ile-Ife, Osun State; University of Ilorin Teaching Hospital, Ilorin, Kwara State, and Babcock University Teaching Hospital, Ilishan-Remo, Ogun State. All raw reads of the clinical isolates are available in the European Nucleotide Archive (https://www.ebi.ac.uk/ena) with study accession number PRJEB29739. Antimicrobial susceptibility data obtained using the same methodology as described for the wastewater isolates were available for 79 of the clinical isolates and were included in the analyses. The remaining 10 clinical isolates could not be resuscitated for repeat AST.

### Bioinformatics analyses

*De novo* genome assembly, species identification, and quality assessment of the generated assemblies were performed using the *de novo* assembly pipeline described in detail in the Genomic Surveillance of Antimicrobial Resistance (GHRU) Retrospective 1 Bioinformatics Methods version 4 (https://www.protocols.io/view/ghru-genomic-surveillance-of-antimicrobial-resista-bp2l6b11kgqe/v4). Only assemblies with N50 values greater than 15,000 and no more than 400 contigs were included in downstream analyses. Contamination was assessed using Confindr, and genomes containing >5% contaminating single-nucleotide variants of selected core genes were also excluded from downstream analyses.

To estimate phylogenetic relationships between the wastewater and clinical isolates, we included the genomes of the 89 previously characterized clinical *A. baumannii* isolates. All genomes were first annotated using Bakta (42) version 1.6.1, after which core genes were identified and aligned using Panaroo (43) version 1.3.2. The filtered core gene alignment, which excluded outlying genes identified based on the Tukey outlier test, was then used as input into RAxML-NG (44) version 1.1.0 for phylogenetic tree inference with the GTR + G model, 50 distinct starting trees, and “bootstopping.” Bootstrapping converged after 300 replicates. To determine the genetic similarity between genomes in specific clades of interest, we selected close reference genomes for each clade from the RefSeq database [accessions: GCF_001908295.1 (clade A), GCF_000828935.1 (clade B), GCF_000828935.1: (clade C), and GCF_000021245.2 (clade D)] and reconstructed clade-specific reference-based phylogenies using the GHRU mapping-based phylogeny pipeline (https://www.protocols.io/view/ghru-genomic-surveillance-of-antimicrobial-resista-bp2l6b11kgqe/v4). Single-nucleotide polymorphism (SNP) distances were determined from the resulting alignments using snp-dists version 0.8.2.

To characterize the population structure and identify possible transmission pairs between clinical and wastewater *A. baumannii* isolates, a *t*-distributed two-dimensional stochastic cluster embedding analysis was conducted to identify hierarchical [Hierarchical Density-based Spatial Clustering of Applications with Noise (HDBSCAN)] clusters using Mandrake (45) version 1.2.2 with the gene presence/absence matrix output from the Panaroo program as input and the following parameters: kNN = 100, perplexity = 40, and maxIter = 300,000,000.

Multi-locus sequence types (MLST) were predicted from the raw reads using the ARIBA (46) software version 2.14.4 with the *A. baumannii* Oxford (47) and Pasteur (48) typing schemes in the PubMLST database. Novel allele sequences were extracted from the assemblies using MLSTar (49) version 0.1.5, and the novel alleles and profiles were submitted to the PubMLST database (50) for allele and sequence type (ST) assignment. Using the BURST software within the PubMLST database, sequence types were assigned to one of the nine classified international clones (ICs) if they had no more than two locus variations from representatives of each clone (14, 51, 52). Antimicrobial resistance genes were detected using AMRfinderplus (53) version 3.10.24 with database version 2022–04-04. Only genes in the “core” database and with an “element type” of “AMR” were included in the analyses. “Partial” hits (<90% of the length of the reference database sequence) determined to not be truncated by a contig boundary were also excluded. The intrinsic *bla*_OXA-51_-like and *bla*_ADC_-like genes were also not reported. To determine the genetic contexts of the detected carbapenem resistance genes, we mapped both the assemblies and the reads of the wastewater isolates to previously characterized mobile genetic elements carrying acquired carbapenem resistance genes in *A. baumannii* (16, 19, 54 – 56) using Gview server (https://server.gview.ca/) with default parameters (for the assemblies) and BWA MEM (57) version 0.7.17 (for the reads). Duplicate reads were marked and removed using Picard version 3.0.0 (http://broadinstitute.github.io/picard).

### Data analyses

Statistical analyses and data visualizations were carried out using R version 4.2.1. The resistance rates to each antimicrobial were compared between the clinical and wastewater *A. baumannii* isolates using Pearson’s chi-squared test with false discovery rate correction for multiple testing. The Wilcoxon rank-sum test with false discovery rate correction was used to compare the number of resistance genes conferring resistance to unique antimicrobial classes between strains with and without at least one carbapenem resistance gene. *P* values less than 0.05 were considered statistically significant.

## RESULTS

### Isolate collection

A total of 24 wastewater samples (12 untreated and 12 treated) were collected for the study. A total of 90 isolates from treated and untreated wastewater samples (range: 0–16 isolates per sample) were identified as *A. baumannii* complex using the VITEK 2 system and subjected to Illumina sequencing. Overall, at least one isolate belonging to the *A. baumannii* complex was recovered in all the months of sampling except June 2021. *A. baumannii* complex isolates were recovered from untreated wastewater samples in 10 of the 12 months of sampling and in eight of the 12 months from the treated wastewater samples. Eighty-two of the 90 sequenced isolates passed the sequence quality checks and were included in the analyses. Based on the whole-genome sequences, 77 of the 82 isolates were identified as *A. baumannii*, while the remaining 5 isolates were *Acinetobacter pittii*. Of the confirmed 77 *A*. *baumannii* isolates, 33 were isolated from the raw/untreated wastewater, while 44 were isolated from the treated effluent.

### Sequence type and lineage distribution

**FIG 1:**
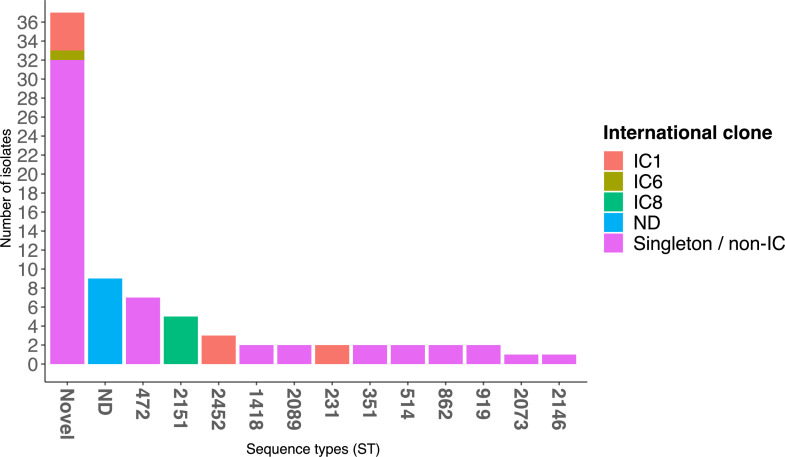
Sequence type distribution of *A. baumannii* isolates from raw and treated wastewater, University College Hospital, Ibadan, Nigeria. IC, international clone; ND, not determined.

Of the 77 *A*. *baumannii* isolates, about half or 37 (48.1%) belonged to 19 novel Oxford STs (Fig. 1). The novel STs were submitted to the PubMLST database and assigned ST numbers. They include ST2828 (*n* = 6), ST2797 (*n* = 4), ST2836 (*n* = 4), ST2803 (*n* = 3), ST2479 (*n* = 2), ST2798 (*n* = 2), ST2827 (*n* = 2), ST2834 (*n* = 2), ST2835 (*n* = 2), ST2796 (*n* = 1), ST2799 (*n* = 1), ST2800 (*n* = 1), ST2801 (*n* = 1), ST2802 (*n* = 1), ST2824 (*n* = 1), ST2829 (*n* = 1), ST2830 (*n* = 1), ST2831 (*n* = 1), and ST2832 (*n* = 1). Other STs detected included ST472 (*n* = 7), ST2151 (*n* = 5), ST2452 (*n* = 4), ST1418 (*n* = 2), ST2089 (*n* = 2), ST231 (*n* = 2), ST351 (*n* = 2), ST514 (*n* = 2), ST862 (*n* = 2), ST919 (*n* = 2), ST2073 (*n* = 1), and ST2146 (*n* = 1). The Oxford STs of nine isolates could not be determined as they were missing at least one of the seven typing loci. Five of these isolates were typed as Pasteur ST203, Pasteur ST1464, Pasteur novel ST2240 (*n* = 2), and Pasteur novel ST2242. Most of the isolates (53/77, 68.8%) belonged to none of the nine known ICs. There were, however, nine isolates belonging to IC1 (ST231, ST2452, and novel ST2797), five belonging to IC8 (ST2151), and one belonging to IC6 (novel ST2830).

**FIG 2:**
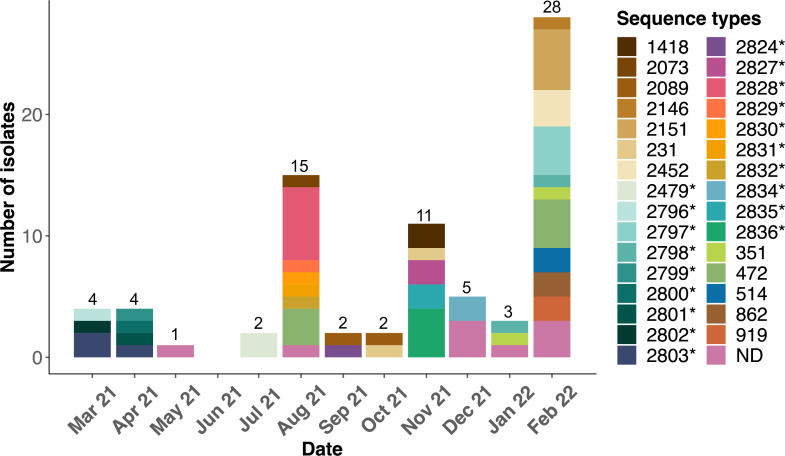
Sequence type distribution of wastewater *A. baumannii* isolates according to date of isolation. *Novel sequence type.

Most of the STs were detected only transiently throughout the study period. Of the 31 distinct STs detected, most (25/31, 78.1%) were detected in only one of the 12 sampling months (Fig. 2). Five of the remaining six STs were recovered in two successive months (ST2803, March and April 2021; ST2089, September and October 2021; ST231, October and November 2021; ST2798, January and February 2022; and ST351, January and February 2022). ST472 was the only ST detected in two non-successive months (August 2021 and February 2022). Six of the 31 distinct STs were detected in raw wastewater but not in treated wastewater; 4 of these were detected only once.

### Phenotypic antimicrobial resistance and antimicrobial resistance genes

**FIG 3:**
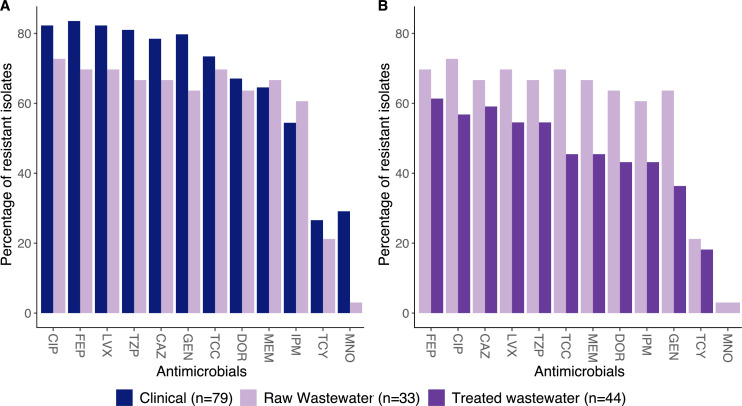
Comparison of phenotypic antimicrobial resistance rates between isolates from (**A**) clinical samples and raw hospital wastewater and (**B**) raw hospital wastewater and treated hospital wastewater. CAZ, ceftazidime; CIP, ciprofloxacin; DOR, doripenem; FEP, cefepime; GEN, gentamicin; IPM, imipenem; LVX, levofloxacin; MEM, meropenem; MNO, minocycline; TCC, ticarcillin/clavulanic acid; TCY, tigecycline; TZP, piperacillin/tazobactam.

At least 50% of the 77 *A*. *baumannii* isolates were resistant to 9 of the 12 tested antimicrobials, including cefepime (*n* = 50, 64.9%), ciprofloxacin (*n* = 49, 63.6%), ceftazidime (*n* = 48, 62.3%), levofloxacin (*n* = 47, 61.0%), piperacillin/tazobactam (*n* = 46, 59.7%), ticarcillin/clavulanic acid (*n* = 43, 55.8%), meropenem (*n* = 42, 54.5%), doripenem (*n* = 40, 51.9%), and imipenem (*n* = 39, 50.6%). The resistance rate to gentamicin was slightly lower (*n* = 37, 48.1%), while 15 isolates (19.5%) were resistant to tigecycline, and one isolate (1.3%) was resistant to minocycline. Forty-two isolates (54.5%) were phenotypically resistant to at least one carbapenem (meropenem, imipenem, or doripenem). All five *A. pittii* isolates were resistant to ceftazidime and cefepime, while three of these strains were resistant to piperacillin/tazobactam. Although the raw wastewater isolates had higher resistance rates to all tested antimicrobials compared to the treated wastewater isolates, these differences were not statistically significant (Fig. 3).

**FIG 4:**
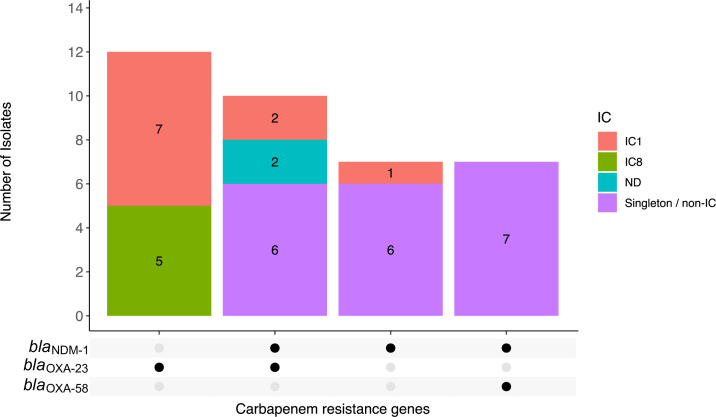
Distribution of carbapenem resistance genes among *A. baumannii* isolated from raw and treated hospital wastewater. The black circles denote gene presence. IC, international clone; ND, not determined.

All hospital wastewater isolates carried at least one aminoglycoside resistance gene. Quinolone resistance-conferring mutations were detected in 42 (54.5%) isolates, including 33 with both *gyrA*_S81L and *parC*_S84L, 6 with *gyrA*_S81L and *parC*_S84F, 2 with *gyrA*_S81L, and 1 with *parC*_S84L. Thirty-six isolates (46.8%) carried at least one acquired carbapenem resistance gene. *bla*_NDM-1_ was the most common (24/77, 31.2%) acquired carbapenem resistance gene detected among the wastewater *A. baumannii* isolates (Fig. 4). This was followed closely by *bla*_OXA-23_ (*n* = 22, 28.6%) and *bla*_OXA-58_ (*n* = 7, 9.1%). Ten of the 24 *bla*_NDM-1_-positive isolates co-carried *bla*_OXA-23_, while another 7 co-carried *bla*_OXA-58_. The *bla*_OXA-23_ genes in all 22 isolates were carried on a Tn*2006* transposon (Fig. S1), while among the 24 *bla*_NDM-1_-positive isolates, 20 carried the gene on the ~10-kb Tn*125* transposon (Fig. S2). The genetic context of the *bla*_NDM-1_ gene in the remaining four isolates (all ST472) was similar to the Tn*7382* transposon but was missing the downstream *dsbD* and *cutA* genes, and possibly the IS*Aba14* insertion sequence (Fig. S2). The exact structure and composition of this transposon could not be determined due to the limitations of the available short-read sequence data. Genes conferring resistance to sulfonamides (44.2%), bleomycin (29.9%), macrolides (22.1%), tetracyclines (20.8%), chloramphenicol (19.5%), trimethoprim (15.6%), and rifamycin (11.7%) were also detected in at least 10% of the isolates. The tigecycline resistance gene *tet(X3*) was present in only the four novel ST2836 isolates. No known colistin resistance-conferring gene or mutation was detected in the isolates’ genomes. Isolates from the final effluent carried fewer genes conferring resistance to unique antimicrobial classes (median = 2 classes) compared to raw wastewater isolates (median = 4 classes). Similarly, the proportions of final treated wastewater isolates that carried at least one carbapenem resistance gene [38.6% (17/44) versus 57.6% (19/33)] and were phenotypically resistant to at least one carbapenem [45.5% (20/44) versus 66.7% (22/33)] were lower compared to the raw wastewater isolates. All these differences were, however, not statistically significant.

**FIG 5:**
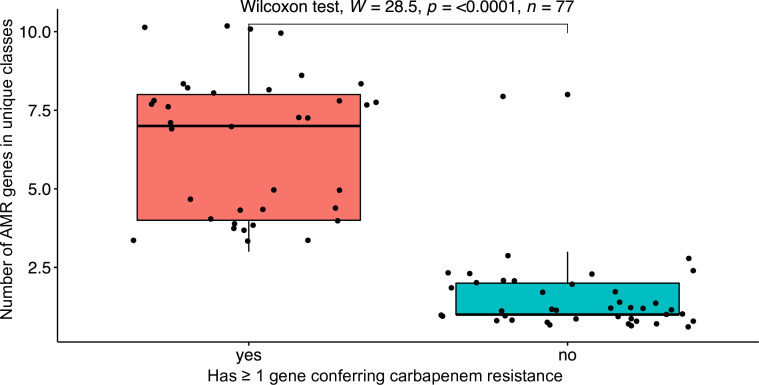
Comparison of the number of genes conferring resistance to unique antimicrobial classes in *A. baumannii* isolates with at least one carbapenem resistance gene versus isolates without a carbapenem resistance gene. AMR, antimicrobial resistance.

Among the 24 *bla*_NDM-1_-positive and 22 *bla*_OXA-23_-positive isolates, 11 and 12, respectively, were from treated wastewater. Isolates with at least one acquired carbapenem resistance gene carried more genes conferring resistance to distinct antimicrobial classes [median = 7 classes; interquartile range (IQR) = 4] compared to isolates without an acquired carbapenem resistance gene (median = 1 class, IQR = 4) (Wilcoxon rank-sum test: adjusted *P* < 0.0001; Fig. 5). Among the 22 isolates carrying *bla*_OXA-23_, 9 belonged to IC1 (ST231, ST2452, and ST2797) and 5 belonged to IC8 (ST2151). The remaining *bla*_OXA-23_-positive isolates belonged to either ST514 (*n* = 3), ST862 (*n* = 2), or ST919 (*n* = 2), or were undetermined; the undetermined STs were closely related to either ST514 or ST862. The *bla*_NDM-1_ genes were similarly distributed, being detected mostly among strains within phylogenetically distinct clades.

### Phylogenetic relatedness between the wastewater and clinical isolates

**FIG 6:**
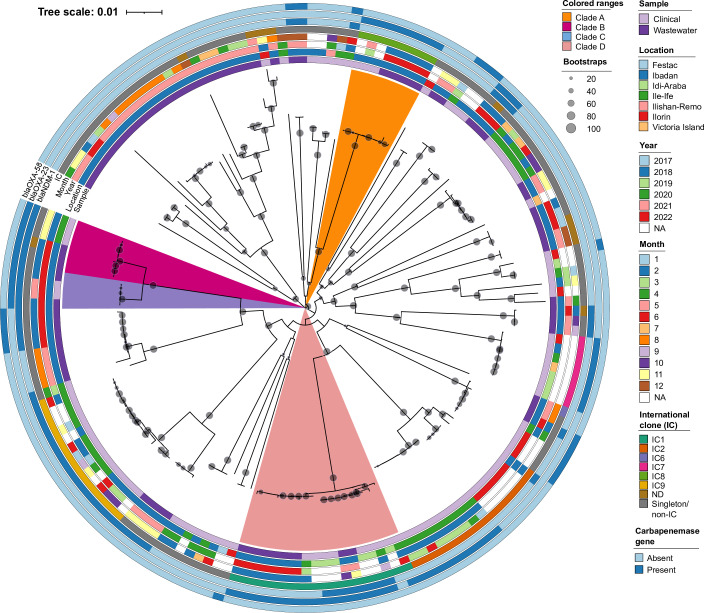
Maximum likelihood phylogeny of 77 *A*. *baumannii* isolates obtained from hospital wastewater between March 2021 and February 2022, and 89 *A*. *baumannii* isolates obtained from various clinical samples between 2016 and 2020. IC, international clone; NA, not available.

To determine whether the wastewater isolates were genetically related to clinical isolates, we constructed a maximum likelihood phylogeny of the wastewater isolates and 89 previously characterized clinical isolates from across southwestern Nigeria. The *A. baumannii* isolates recovered from wastewater were highly phylogenetically diverse and occupied distinct, deeply branching clades (Fig. 6). There were 28 distinct HDBSCAN clusters (clusters 0–27) generated from the pangenome gene presence/absence matrix using Mandrake, and these mostly correlated with the distinct clades on the phylogenetic tree. In general, there were a few phylogenetic and/or HDBSCAN clusters of clinical and wastewater isolates. Five *bla*_OXA-23_-carrying ST2151 (IC8) isolates obtained from raw (*n* = 3) and treated (*n* = 2) wastewater in February 2022 clustered together (cluster 17) with three clinical isolates from the same healthcare facility, one of which was isolated from blood in May 2019 (clade A). Despite this clustering, these three clinical isolates, which were all identical (0 SNPs), were not phylogenetically identical to any of the five wastewater isolates (between 603 and 953 SNPs). Conversely, three isolates (two ST862 and one non-typeable ST) also recovered from raw and treated wastewater in February 2022 were phylogenetically similar (30–66 SNPs) to three blood isolates that were isolated back in November 2018 in a neighboring city, Ile-Ife, Osun State (clade B). All six wastewater and clinical strains co-carried *bla*_NDM-1_ and *bla*_OXA-23_.

Multiple isolates from final treated wastewater were phylogenetically identical to other isolates from raw (untreated) wastewater and to clinical isolates. Two ST919 isolates from treated wastewater in February 2022 carrying both *bla*_NDM-1_ and *bla*_OXA-23_ were phylogenetically identical (differed by two to four SNPs) to two clinical isolates subsequently isolated from a tracheal aspirate sample of a patient in the same healthcare facility in May 2022 (clade C). Within clade D, which comprised the different IC1 isolates, there were two sub-clades containing clusters of isolates from clinical and wastewater samples. The first sub-clade (cluster 4, ST231) included five clinical isolates that were closely related to two isolates from raw and treated wastewater. All seven isolates carried both *bla*_NDM-1_ and *bla*_OXA-23_. The other sub-clade comprised two clinical isolates from blood, three isolates from raw hospital wastewater, and four isolates from treated wastewater, all of which carried *bla*_OXA-23_ and belonged to HDBSCAN cluster 3.

In terms of antimicrobial resistance, the clinical *A. baumannii* isolates had higher resistance rates than almost all the tested antimicrobials (except imipenem and meropenem) compared to the 33 isolates from raw wastewater, but these differences were not statistically significant (Fig. 3).

## DISCUSSION

Wastewater treatment plants, particularly those that receive clinical waste, are known to be important sources of drug-resistant pathogens, including *A. baumannii* (25). In this study, we investigated treated hospital wastewater as a source of clinically relevant *A. baumannii* strains. *A. baumannii* isolates were recovered throughout the 1-year study period, consistent with previous reports of the high prevalence of *A. baumannii* in hospital wastewater elsewhere (22, 25, 58). It is uncertain to what extent the reported chlorination of the treated wastewater before discharge impacted occurrence and resistance levels in *A. baumannii*.

Notably, there was a high diversity of *A. baumannii* lineages in hospital wastewater, most of which belonged to novel or sparsely described STs. A few STs belonged to the major international clones IC1, IC6, and IC9, but the majority were either singletons or belonged to none of the globally disseminated clones. Despite the recovery of *A. baumannii* complex isolates in all but 1 of the 12 sampling months, specific *A. baumannii* sequence types were only detected transiently throughout the study, suggesting the absence of a reservoir for these STs in the hospital environment and reflecting the previously identified diversity of *A. baumannii* lineages in clinical settings in southwestern Nigeria (19). Interestingly, no strain belonging to IC2, which is the most predominant clinical *A. baumannii* lineage in Nigeria (19) and has been previously reported in hospital wastewater in Germany (25), was detected throughout the 12 months.

The detection of so many novel STs in hospital wastewater may indicate missed/unreported infections or hospital environment or patient colonization. *A. baumannii* spp. mostly exist as hospital environment or human colonizers and cause infections primarily in immunocompromised patients (5, 59, 60), but colonization in itself is associated with increased risk of subsequent infection among patients (14, 61). One interesting finding was the detection of two isolates in treated wastewater in February 2022 that were nearly identical to two clinical isolates from a hospitalized patient in the same hospital 3 months later in May 2022. These four ST919 wastewater and clinical strains were clonal, differing by four to nine SNPs, and all carried both *bla*_NDM-1_ and *bla*_OXA-23_. The two wastewater isolates were both resistant to cefepime, ceftazidime, ciprofloxacin, doripenem, imipenem, meropenem, levofloxacin, piperacillin/tazobactam, and ticarcillin/clavulanic acid, but there were no antimicrobial susceptibility data for the clinical isolates. Based on the available data, we cannot determine the association or direction of transmission between these strains, but they illustrate the likely existence of *A. baumannii* transmission links between the hospital environment and the human population.

We identified a few phylogenetic and/or HDBSCAN clusters of clinical and wastewater isolates, indicating the potential clinical significance and likely clinical source of the wastewater isolates. The low numbers of these clusters may be due to the preponderance of novel STs among the wastewater isolates and the fact that *Acinetobacter* spp. causing clinical infections are likely under-detected in our setting (19). Importantly, the clustering and close phylogenetic relationships between the clinical and wastewater isolates, including those from the final treated effluent, illustrate the presence of possible transmission chains from the hospital environment or even patients to the environment receiving the final treated wastewater. This release of *A. baumannii* into the environment via treated wastewater represents a significant public health concern requiring intervention as there is evidence that clinically relevant *A. baumannii* can persist in low-nutrient and even oxygen-deprived environments of the receiving water bodies for up to 50 days, which is potentially long enough to reach the human population (23, 29). The subsequent transmission of carbapenemase-producing strains via lotic water has also been demonstrated previously (62). More so, the likelihood of these transmissions and their public health implications are even worse in low- and middle-income regions where poor water sanitation and hygiene conditions pervade and transmission routes are abundant (63, 64).

Antimicrobial resistance rates were high among the wastewater *A. baumannii* isolates, with at least 50% resistance reported for 9 of the 12 tested antimicrobials. The continued spread of carbapenem-resistant *A. baumannii* is a huge health challenge globally as the remaining treatment options for these strains are severely limited due to resistance, *in vivo* efficacy, toxicity, cost, and pharmacokinetic issues associated with the other recommended therapeutic options such as the polymyxins, tetracyclines, and sulbactam (11, 20, 21). Carbapenemase gene carriage, which we determined to be significantly linked to carriage of multiple antimicrobial resistance determinants, was also notably high among the wastewater isolates. This is consistent with previous observations that carbapenem-resistant *A. baumannii* are often resistant to multiple antimicrobial classes (21). The exact reason for this phenomenon is unclear, but a possible reason is the fact that plasmids, large genomic islands, and other mobile genetic elements carrying carbapenem resistance genes in *A. baumannii* are often found associated with genes conferring resistance to multiple other antimicrobial classes (65). It is noteworthy that the rates of resistance to carbapenems and other antimicrobials and the number of resistance genes were lower among the final effluent isolates compared to isolates from raw wastewater, which is consistent with previous reports that the proportion of resistant isolates is likely to be reduced in final treated wastewater (23, 25). Nevertheless, the carbapenem resistance rate observed among final effluent isolates in this study was still notably high (45.5%). The release of carbapenem-resistant *A. baumannii* into the environment via hospital wastewater may play a role in the continued spread of drug-resistant *A. baumannii*, and the relative importance of wastewater as a source for transmission and exposure to humans needs to be assessed. A recent study investigating the presence of multi-drug-resistant *A. baumannii* in various livestock and human wastewater sources found that only those from hospitals contained multi-drug-resistant isolates of *A. baumannii*, suggesting a lesser role of the livestock industry in the spread of these clinically relevant strains (25).

This study confirms the increasing prevalence of *bla*_NDM-1_ among *A. baumannii* isolates reported in recent studies, primarily in Africa and the Middle East (17, 19, 66 – 69). About a third of the wastewater isolates carried the *bla*_NDM-1_ gene, and almost half of these were isolates from the final treated wastewater released into the environment. The distribution of *bla*_NDM-1_ and *bla*_OXA-23_ predominantly among isolates in distinct phylogenetic clusters suggests that clonal expansion of carbapenem-resistant clones is the primary driver of increasing carbapenem resistance prevalence among *A. baumannii*. This is consistent with previous reports, and the clonal spread of carbapenem-resistant *A. baumannii* lineages both geographically and within hospital settings is well described in the literature (8, 70 – 76). Nevertheless, the seemingly lower frequency of horizontal acquisition of carbapenem resistance genes is still interesting as *A. baumannii* spp. have highly plastic genomes and a high tendency to acquire resistance genes on plasmids and other mobile genetic elements like transposons (3, 8, 77). One explanation for this may be the frequent carriage of carbapenem resistance genes on chromosomes by *A. baumannii*. In our previous study characterizing clinical *A. baumannii* isolates in the southwestern region of Nigeria, in all the isolates where the location of *bla*_NDM-1_ and *bla*_OXA-23_ genes were determined, both genes were chromosomally located (19). In these clinical strains, *bla*_OXA-23_ was carried entirely on Tn*2006* or Tn*2006*-like transposons, as observed for the wastewater isolates in this study. Similarly, 20 of the 24 *bla*_NDM-1_-positive wastewater isolates (belonging to nine distinct STs) carried the gene on the Tn*125* transposon, which is a highly mobilizable transposon believed to be the primary means of horizontal dissemination of *bla*_NDM-1_ among *A. baumannii* and other Gram-negative pathogens (19, 56, 78). Wastewater contains sub-inhibitory concentrations of antimicrobials and is regarded as a potential hotspot for the increased exchange and uptake of antimicrobial resistance genes, and could thus accelerate the horizontal spread of these genes and mobile elements both between *A. baumannii* and to other species (79). Further studies are needed to understand the dynamics of carbapenem resistance gene acquisition among *A. baumannii* lineages and other bacterial populations in wastewater and related aquatic environments.

### Limitations

The monthly samples and lack of a composite sampling technique preclude any definitive conclusions on the longitudinal trends and incidence of carbapenem-resistant *A. baumannii* in raw and treated wastewater in the facility. Similarly, due to the lack of an efficient method for the selective recovery of *A. baumannii* isolates from water samples, as has been previously described (25), we could not meaningfully interpret, and thus present, isolate count data, which would have been a useful denominator. We included 2 µg/mL of cefotaxime in the primary isolation plates to facilitate the selective isolation of *A. baumannii*, but both beta-lactam-resistant and beta-lactam-sensitive *A. baumannii* isolates, as well as non-target species, were recovered throughout, a phenomenon that is common during *A. baumannii* isolation even with higher concentrations of antibiotic supplements (22, 25). Despite this, the cefotaxime supplement may have overestimated the reported beta-lactam resistance rates. Furthermore, the primary plates recovered isolates with morphologies similar to *Acinetobacter baumannii* complex isolates that were subsequently determined to belong to other species, including *Aeromonas hydrophila*, *Pseudomonas putida*, *Pseudomonas stutzeri*, and *Stenotrophomonas maltophila*. Thus, plate counts could not be reliably reported as *A. baumannii* counts. Another limitation was our inability to concurrently obtain a larger number of clinical isolates from the same hospital that would have allowed more robust interpretations of the transmission links between the hospital wards and the environment. Finally, as only wastewater from one health facility was sampled, these results cannot be generalized to other health facilities in Nigeria.

### Conclusions

Carbapenem-resistant *A. baumannii* strains belonging to diverse lineages can survive the hospital wastewater treatment process and can be released into the environment. This necessitates the improvement of hospital wastewater treatment processes to more effectively eliminate these pathogens. The importance of such treated wastewater as a contributor to the increasing prevalence of *A. baumannii* lineages carrying carbapenem resistance genes is uncertain and needs further studies. Addressing this challenge requires interventions guided by a robust genomic surveillance of these high-priority pathogens using a One Health approach.

## Data Availability

The raw reads of all sequenced wastewater *A. baumannii* genomes have been deposited in the European Nucleotide Archive with study accession number PRJEB58695. The individual sample accession numbers, metadata, and raw analysis data are also available from this Microreact project (https://microreact.org/project/ortEjJKjw8YRmdbnZVdMFC-wastewater-and-clinical-a-baumannii-core-genome-phylogeny-2022-12-30).

## SUPPLEMENTAL MATERIAL

Supplemental figures spectrum.02381-23-s0001.docx Fig. S1 and S2.

ASM does not own the copyrights to Supplemental Material that may be linked to, or accessed through, an article. The authors have granted ASM a non-exclusive, world-wide license to publish the Supplemental Material files. Please contact the corresponding author directly for reuse.

## References

[1] Jawad A , Seifert H , Snelling AM , Heritage J , Hawkey PM . 1998. Survival of Acinetobacter baumannii on dry surfaces: comparison of outbreak and sporadic isolates. J Clin Microbiol 36:1938–1941. doi:10.1128/JCM.36.7.1938-1941.1998 9650940 PMC104956

[2] Obeidat N , Jawdat F , Al-Bakri AG , Shehabi AA . 2014. Major biologic characteristics of Acinetobacter baumannii isolates from hospital environmental and patients’ respiratory tract sources. Am J Infect Control 42:401–404. doi:10.1016/j.ajic.2013.10.010 24679567

[3] Peleg AY , Seifert H , Paterson DL . 2008. Acinetobacter baumannii: emergence of a successful pathogen. Clin Microbiol Rev 21:538–582. doi:10.1128/CMR.00058-07 18625687 PMC2493088

[4] Harding CM , Hennon SW , Feldman MF . 2018. Uncovering the mechanisms of Acinetobacter baumannii virulence. Nat Rev Microbiol 16:91–102. doi:10.1038/nrmicro.2017.148 29249812 PMC6571207

[5] Chia PY , Sengupta S , Kukreja A , S L Ponnampalavanar S , Ng OT , Marimuthu K . 2020. The role of hospital environment in transmissions of multidrug-resistant Gram-negative organisms. Antimicrob Resist Infect Control 9:29. doi:10.1186/s13756-020-0685-1 32046775 PMC7014667

[6] Umezawa K , Asai S , Ohshima T , Iwashita H , Ohashi M , Sasaki M , Kaneko A , Inokuchi S , Miyachi H . 2015. Outbreak of drug-resistant Acinetobacter baumannii ST219 caused by oral care using tap water from contaminated hand hygiene sinks as a reservoir. Am J Infect Control 43:1249–1251. doi:10.1016/j.ajic.2015.06.016 26388038

[7] Karakonstantis S , Gikas A , Astrinaki E , Kritsotakis EI . 2020. Excess mortality due to pandrug-resistant Acinetobacter baumannii infections in hospitalized patients. J Hosp Infect 106:447–453. doi:10.1016/j.jhin.2020.09.009 32927013

[8] Hamidian M , Nigro SJ . 2019. Emergence, molecular mechanisms and global spread of carbapenem-resistant Acinetobacter baumannii. Microb Genom 5:e000306. doi:10.1099/mgen.0.000306 31599224 PMC6861865

[9] Medioli F , Bacca E , Faltoni M , Burastero GJ , Volpi S , Menozzi M , Orlando G , Bedini A , Franceschini E , Mussini C , Meschiari M . 2022. Is it possible to eradicate carbapenem-resistant Acinetobacter baumannii (CRAB) from endemic hospitals? Antibiotics 11:1015. doi:10.3390/antibiotics11081015 36009885 PMC9405503

[10] Tacconelli E , Carrara E , Savoldi A , Harbarth S , Mendelson M , Monnet DL , Pulcini C , Kahlmeter G , Kluytmans J , Carmeli Y , Ouellette M , Outterson K , Patel J , Cavaleri M , Cox EM , Houchens CR , Grayson ML , Hansen P , Singh N , Theuretzbacher U , Magrini N , Aboderin AO , Al-Abri SS , Awang Jalil N , Benzonana N , Bhattacharya S , Brink AJ , Burkert FR , Cars O , Cornaglia G , Dyar OJ , Friedrich AW , Gales AC , Gandra S , Giske CG , Goff DA , Goossens H , Gottlieb T , Guzman Blanco M , Hryniewicz W , Kattula D , Jinks T , Kanj SS , Kerr L , Kieny M-P , Kim YS , Kozlov RS , Labarca J , Laxminarayan R , Leder K , Leibovici L , Levy-Hara G , Littman J , Malhotra-Kumar S , Manchanda V , Moja L , Ndoye B , Pan A , Paterson DL , Paul M , Qiu H , Ramon-Pardo P , Rodríguez-Baño J , Sanguinetti M , Sengupta S , Sharland M , Si-Mehand M , Silver LL , Song W , Steinbakk M , Thomsen J , Thwaites GE , van der Meer JW , Van Kinh N , Vega S , Villegas MV , Wechsler-Fördös A , Wertheim HFL , Wesangula E , Woodford N , Yilmaz FO , Zorzet A . 2018. Discovery, research, and development of new antibiotics: the WHO priority list of antibiotic-resistant bacteria and tuberculosis. Lancet Infect Dis 18:318–327. doi:10.1016/S1473-3099(17)30753-3 29276051

[11] Fishbain J , Peleg AY . 2010. Treatment of acinetobacter infections. Clin Infect Dis 51:79–84. doi:10.1086/653120 20504234

[12] Lowe M , Singh-Moodley A , Ismail H , Thomas T , Chibabhai V , Nana T , Lowman W , Ismail A , Chan WY , Perovic O . 2022. Molecular characterisation of Acinetobacter baumannii isolates from bloodstream infections in a tertiary-level hospital in South Africa. Front Microbiol 13:863129. doi:10.3389/fmicb.2022.863129 35992699 PMC9391000

[13] Orhan Ö , Yılmaz K , Gözü Pirinççioğlu A , Solmaz M , Karakoç F . 2022. Antibiotic susceptibility of microorganisms grown in tracheal aspirate cultures of pediatric intensive care patients. Cureus 14:e26934. doi:10.7759/cureus.26934 35989826 PMC9379867

[14] Odih EE , Irek EO , Obadare TO , Oaikhena AO , Afolayan AO , Underwood A , Adenekan AT , Ogunleye VO , Argimon S , Dalsgaard A , Aanensen DM , Okeke IN , Aboderin AO . 2022. Rectal colonization and nosocomial transmission of carbapenem-resistant Acinetobacter baumannii in an intensive care unit, Southwest Nigeria. Front Med 9:846051. doi:10.3389/fmed.2022.846051 PMC8936076 35321470

[15] Makke G , Bitar I , Salloum T , Panossian B , Alousi S , Arabaghian H , Medvecky M , Hrabak J , Merheb-Ghoussoub S , Tokajian S . 2020. Whole-genome-sequence-based characterization of extensively drug-resistant Acinetobacter baumannii hospital outbreak. mSphere 5:e00934-19. doi:10.1128/mSphere.00934-19 31941816 PMC6968657

[16] Nigro SJ , Hall RM . 2018. Does the intrinsic oxaAb (blaOXA-51-like) gene of Acinetobacter baumannii confer resistance to carbapenems when activated by ISAba1? J Antimicrob Chemother 73:3518–3520. doi:10.1093/jac/dky334 30124881

[17] Yehouenou C , Bogaerts B , Vanneste K , Roosens NHC , De Keersmaecker SCJ , Marchal K , Affolabi D , Soleimani R , Rodriguez-Villalobos H , Van Bambeke F , Dalleur O , Simon A . 2021. First detection of a plasmid-encoded New-Delhi metallo-beta-lactamase-1 (NDM-1) producing Acinetobacter baumannii using whole genome sequencing, isolated in a clinical setting in Benin. Ann Clin Microbiol Antimicrob 20:5. doi:10.1186/s12941-020-00411-w 33407536 PMC7789245

[18] Wang J , Ning Y , Li S , Wang Y , Liang J , Jin C , Yan H , Huang Y . 2019. Multidrug‑resistant Acinetobacter baumannii strains with NDM‑1: molecular characterization and in vitro efficacy of meropenem‑based combinations. Exp Ther Med 18:2924–2932. doi:10.3892/etm.2019.7927 31572535 PMC6755477

[19] Odih EE , Oaikhena AO , Underwood A , Hounmanou YMG , Oduyebo OO , Fadeyi A , Aboderin AO , Ogunleye VO , Argimón S , Akpunonu VN , Oshun PO , Egwuenu A , Okwor TJ , Ihekweazu C , Aanensen DM , Dalsgaard A , Okeke IN . 2023. High genetic diversity of carbapenem-resistant Acinetobacter baumannii isolates recovered in Nigerian hospitals in 2016 to 2020. mSphere 8:e0009823. doi:10.1128/msphere.00098-23 37067411 PMC10286719

[20] Isler B , Doi Y , Bonomo RA , Paterson DL . 2019. New treatment options against carbapenem-resistant Acinetobacter baumannii infections. Antimicrob Agents Chemother 63:e01110-18. doi:10.1128/AAC.01110-18 PMC6325237 30323035

[21] O’Donnell JN , Putra V , Lodise TP . 2021. Treatment of patients with serious infections due to carbapenem-resistant Acinetobacter baumannii: how viable are the current options?. Pharmacother J Hum Pharmacol Drug Ther 41:762–780. doi:10.1002/phar.2607 34170571

[22] Higgins PG , Hrenovic J , Seifert H , Dekic S . 2018. Characterization of Acinetobacter baumannii from water and sludge line of secondary wastewater treatment plant. Water Res 140:261–267. doi:10.1016/j.watres.2018.04.057 29723815

[23] Hubeny J , Korzeniewska E , Buta-Hubeny M , Zieliński W , Rolbiecki D , Harnisz M . 2022. Characterization of carbapenem resistance in environmental samples and Acinetobacter spp. isolates from wastewater and river water in Poland. Sci Total Environ 822:153437. doi:10.1016/j.scitotenv.2022.153437 35122847

[24] Müller H , Sib E , Gajdiss M , Klanke U , Lenz-Plet F , Barabasch V , Albert C , Schallenberg A , Timm C , Zacharias N , Schmithausen RM , Engelhart S , Exner M , Parcina M , Schreiber C , Bierbaum G . 2018. Dissemination of multi-resistant Gram-negative bacteria into German wastewater and surface waters. FEMS Microbiol Ecol 94. doi:10.1093/femsec/fiy057 29659796

[25] Pulami D , Kämpfer P , Glaeser SP . 2023. High diversity of the emerging pathogen Acinetobacter baumannii and other Acinetobacter spp. in raw manure, biogas plants digestates, and rural and urban wastewater treatment plants with system specific antimicrobial resistance profiles. Sci Total Environ 859:160182. doi:10.1016/j.scitotenv.2022.160182 36395844

[26] Guo J , Li J , Chen H , Bond PL , Yuan Z . 2017. Metagenomic analysis reveals wastewater treatment plants as hotspots of antibiotic resistance genes and mobile genetic elements. Water Res 123:468–478. doi:10.1016/j.watres.2017.07.002 28689130

[27] Obayiuwana A , Ibekwe AM . 2020. Antibiotic resistance genes occurrence in wastewaters from selected pharmaceutical facilities in Nigeria. Water 12:1897. doi:10.3390/w12071897

[28] Adekanmbi AO , Adejoba AT , Banjo OA , Saki M . 2020. Detection of sul1 and sul2 genes in sulfonamide-resistant bacteria (SRB) from sewage, aquaculture sources, animal wastes and hospital wastewater in South-West Nigeria. Gene Rep 20:100742. doi:10.1016/j.genrep.2020.100742

[29] Dekic S , Hrenovic J , van Wilpe E , Venter C , Goic-Barisic I . 2019. Survival of emerging pathogen Acinetobacter baumannii in water environment exposed to different oxygen conditions. Water Sci Technol 80:1581–1590. doi:10.2166/wst.2019.408 31961820

[30] Dexter C , Murray GL , Paulsen IT , Peleg AY . 2015. Community-acquired Acinetobacter baumannii: clinical characteristics, epidemiology and pathogenesis. Expert Rev Anti Infect Ther 13:567–573. doi:10.1586/14787210.2015.1025055 25850806

[31] Xu A , Zhu H , Gao B , Weng H , Ding Z , Li M , Weng X , He G . 2020. Diagnosis of severe community-acquired pneumonia caused by Acinetobacter baumannii through next-generation sequencing: a case report. BMC Infect Dis 20:45. doi:10.1186/s12879-019-4733-5 31941459 PMC6964051

[32] Falagas ME , Karveli EA , Kelesidis I , Kelesidis T . 2007. Community-acquired Acinetobacter infections. Eur J Clin Microbiol Infect Dis 26:857–868. doi:10.1007/s10096-007-0365-6 17701432

[33] Peña-Tuesta I , Del Valle-Vargas C , Petrozzi-Helasvuo V , Aguilar-Luis MA , Carrillo-Ng H , Silva-Caso W , Del Valle-Mendoza J . 2021. Community acquired Acinetobacter baumannii in pediatric patients under 1 year old with a clinical diagnosis of whooping cough in Lima, Peru. BMC Res Notes 14:412. doi:10.1186/s13104-021-05826-y 34758882 PMC8579657

[34] Ong CWM , Lye DCB , Khoo KL , Chua GSW , Yeoh SF , Leo YS , Tambyah PA , Chua AC . 2009. Severe community-acquired Acinetobacter baumannii pneumonia: an emerging highly lethal infectious disease in the Asia–Pacific. Respirology 14:1200–1205. doi:10.1111/j.1440-1843.2009.01630.x 19909464

[35] McCarthy B , Apori SO , Giltrap M , Bhat A , Curtin J , Tian F . 2021. Hospital effluents and wastewater treatment plants: a source of oxytetracycline and antimicrobial-resistant bacteria in seafood. Sustainability 13:13967. doi:10.3390/su132413967

[36] O’Keeffe J . 2021. Wastewater-based epidemiology: current uses and future opportunities as a public health surveillance tool. Environ Health Rev 64:44–52. doi:10.5864/d2021-015

[37] Chau KK , Barker L , Budgell EP , Vihta KD , Sims N , Kasprzyk-Hordern B , Harriss E , Crook DW , Read DS , Walker AS , Stoesser N . 2022. Systematic review of wastewater surveillance of antimicrobial resistance in human populations. Environ Int 162:107171. doi:10.1016/j.envint.2022.107171 35290866 PMC8960996

[38] University College Hospital Ibadan . The University College Hospital Ibadan. Available from: https://uch-ibadan.org.ng. Retrieved 525 SepJanuary 2023. Accessed , 525 SepJanuary 2023

[39] CLSI . 2021. M100Ed32 | performance standards for antimicrobial susceptibility testing. 32nd Ed. Clinical and Laboratory Standards Institute, Wayne, PA. https://clsi.org/standards/products/microbiology/documents/m100.

[40] The European Committee on Antimicrobial Susceptibility Testing . 2022. Breakpoint tables for interpretation of MICs and zone diameters v11.0. https://www.eucast.org/clinical_breakpoints.

[41] Assimakopoulos SF , Karamouzos V , Eleftheriotis G , Lagadinou M , Bartzavali C , Kolonitsiou F , Paliogianni F , Fligou F , Marangos M . 2023. Efficacy of fosfomycin-containing regimens for treatment of bacteremia due to pan-drug resistant Acinetobacter baumannii in critically ill patients: a case series study. Pathogens 12:286. doi:10.3390/pathogens12020286 36839558 PMC9961360

[42] Schwengers O , Jelonek L , Dieckmann MA , Beyvers S , Blom J , Goesmann A . 2021. Bakta: rapid and standardized annotation of bacterial genomes via alignment-free sequence identification. Microb Genom 7:000685. doi:10.1099/mgen.0.000685 34739369 PMC8743544

[43] Tonkin-Hill G , MacAlasdair N , Ruis C , Weimann A , Horesh G , Lees JA , Gladstone RA , Lo S , Beaudoin C , Floto RA , Frost SDW , Corander J , Bentley SD , Parkhill J . 2020. Producing polished prokaryotic pangenomes with the Panaroo pipeline. Genome Biol 21:180. doi:10.1186/s13059-020-02090-4 32698896 PMC7376924

[44] Kozlov AM , Darriba D , Flouri T , Morel B , Stamatakis A . 2019. RAxML-NG: a fast, scalable and user-friendly tool for maximum likelihood phylogenetic inference. Bioinformatics 35:4453–4455. doi:10.1093/bioinformatics/btz305 31070718 PMC6821337

[45] Lees JA , Tonkin-Hill G , Yang Z , Corander J . 2022. Mandrake: visualizing microbial population structure by embedding millions of genomes into a low-dimensional representation. Philos Trans R Soc Lond B Biol Sci 377:20210237. doi:10.1098/rstb.2021.0237 35989601 PMC9393562

[46] Hunt M , Mather AE , Sánchez-Busó L , Page AJ , Parkhill J , Keane JA , Harris SR . 2017. ARIBA: rapid antimicrobial resistance genotyping directly from sequencing reads. Microb Genom 3:e000131. doi:10.1099/mgen.0.000131 29177089 PMC5695208

[47] Bartual SG , Seifert H , Hippler C , Luzon MAD , Wisplinghoff H , Rodríguez-Valera F . 2005. Development of a multilocus sequence typing scheme for characterization of clinical isolates of Acinetobacter baumannii. J Clin Microbiol 43:4382–4390. doi:10.1128/JCM.43.9.4382-4390.2005 16145081 PMC1234098

[48] Diancourt L , Passet V , Nemec A , Dijkshoorn L , Brisse S . 2010. The population structure of Acinetobacter baumannii: expanding multiresistant clones from an ancestral susceptible genetic pool. PLoS One 5:e10034. doi:10.1371/journal.pone.0010034 20383326 PMC2850921

[49] Ferrés I , Iraola G . 2018. MLSTar: automatic multilocus sequence typing of bacterial genomes in R. PeerJ 6:e5098. doi:10.7717/peerj.5098 29922519 PMC6005169

[50] Jolley KA , Bray JE , Maiden MCJ . 2018. Open-access bacterial population genomics: BIGSdb software, the PubMLST.org website and their applications. Wellcome Open Res 3:124. doi:10.12688/wellcomeopenres.14826.1 30345391 PMC6192448

[51] Higgins PG , Prior K , Harmsen D , Seifert H . 2017. Development and evaluation of a core genome multilocus typing scheme for whole-genome sequence-based typing of Acinetobacter baumannii. PLoS One 12:e0179228. doi:10.1371/journal.pone.0179228 28594944 PMC5464626

[52] Xanthopoulou K , Urrutikoetxea-Gutiérrez M , Vidal-Garcia M , Diaz de Tuesta Del Arco J-L , Sánchez-Urtaza S , Wille J , Seifert H , Higgins PG , Gallego L . 2020. First report of New Delhi Metallo-β-lactamase-6 (NDM-6) in a clinical Acinetobacter baumannii isolate from northern Spain. Front Microbiol 11:589253. doi:10.3389/fmicb.2020.589253 33240245 PMC7683408

[53] Feldgarden M , Brover V , Gonzalez-Escalona N , Frye JG , Haendiges J , Haft DH , Hoffmann M , Pettengill JB , Prasad AB , Tillman GE , Tyson GH , Klimke W . 2021. AMRFinderPlus and the reference gene catalog facilitate examination of the genomic links among antimicrobial resistance, stress response, and virulence. Sci Rep 11:12728. doi:10.1038/s41598-021-91456-0 34135355 PMC8208984

[54] Campos JC , da Silva MJF , dos Santos PRN , Barros EM , Pereira M de O , Seco BMS , Magagnin CM , Leiroz LK , de Oliveira TGM , de Faria-Júnior C , Cerdeira LT , Barth AL , Sampaio SCF , Zavascki AP , Poirel L , Sampaio JLM . 2015. Characterization of Tn3000, a transposon responsible for blaNDM-1 dissemination among enterobacteriaceae in Brazil, Nepal, Morocco, and India. Antimicrob Agents Chemother 59:7387–7395. doi:10.1128/AAC.01458-15 26392506 PMC4649174

[55] Hamed SM , Hussein AFA , Al-Agamy MH , Radwan HH , Zafer MM . 2022. Tn7382, a novel composite transposon harboring blaNDM-1 and aphA6 in Acinetobacter baumannii. J Glob Antimicrob Resist 30:414–417. doi:10.1016/j.jgar.2022.08.001 35944804

[56] Poirel L , Bonnin RA , Boulanger A , Schrenzel J , Kaase M , Nordmann P . 2012. Tn125-related acquisition of blaNDM-like genes in Acinetobacter baumannii. Antimicrob Agents Chemother 56:1087–1089. doi:10.1128/AAC.05620-11 22143526 PMC3264265

[57] Li H , Durbin R . 2009. Fast and accurate short read alignment with Burrows-Wheeler transform. Bioinforma Oxf Engl 25:1754–1760. doi:10.1093/bioinformatics/btp324 PMC2705234 19451168

[58] Marathe NP , Berglund F , Razavi M , Pal C , Dröge J , Samant S , Kristiansson E , Larsson DGJ . 2019. Sewage effluent from an Indian hospital harbors novel carbapenemases and integron-borne antibiotic resistance genes. Microbiome 7:97. doi:10.1186/s40168-019-0710-x 31248462 PMC6598227

[59] Asif M , Alvi IA , Rehman SU . 2018. Insight into Acinetobacter baumannii: pathogenesis, global resistance, mechanisms of resistance, treatment options, and alternative modalities. Infect Drug Resist 11:1249–1260. doi:10.2147/IDR.S166750 30174448 PMC6110297

[60] Roca I , Espinal P , Vila-Farrés X , Vila JE . 2012. The Acinetobacter baumannii oxymoron: commensal hospital dweller turned pan-drug-resistant menace. Front Microbiol 3:148. doi:10.3389/fmicb.2012.00148 22536199 PMC3333477

[61] Latibeaudiere R , Rosa R , Laowansiri P , Arheart K , Namias N , Munoz-Price LS . 2015. Surveillance cultures growing carbapenem-resistant Acinetobacter baumannii predict the development of clinical infections: a retrospective cohort study. Clin Infect Dis 60:415–422. doi:10.1093/cid/ciu847 25352586

[62] Laurens C , Jean-Pierre H , Licznar-Fajardo P , Hantova S , Godreuil S , Martinez O , Jumas-Bilak E . 2018. Transmission of IMI-2 carbapenemase-producing enterobacteriaceae from river water to human. J Glob Antimicrob Resist 15:88–92. doi:10.1016/j.jgar.2018.06.022 30279153

[63] Ikhimiukor OO , Odih EE , Donado-Godoy P , Okeke IN . 2022. A bottom-up view of antimicrobial resistance transmission in developing countries. Nat Microbiol 7:757–765. doi:10.1038/s41564-022-01124-w 35637328

[64] Prüss-Ustün A , Wolf J , Bartram J , Clasen T , Cumming O , Freeman MC , Gordon B , Hunter PR , Medlicott K , Johnston R . 2019. Burden of disease from inadequate water, sanitation and hygiene for selected adverse health outcomes: an updated analysis with a focus on low- and middle-income countries. Int J Hyg Environ Health 222:765–777. doi:10.1016/j.ijheh.2019.05.004 31088724 PMC6593152

[65] Dijkshoorn L , Nemec A , Seifert H . 2007. An increasing threat in hospitals: multidrug-resistant Acinetobacter baumannii. Nat Rev Microbiol 5:939–951. doi:10.1038/nrmicro1789 18007677

[66] Fernández-Cuenca F , Pérez-Palacios P , Galán-Sánchez F , López-Cerero L , López-Hernández I , López Rojas R , Arca-Suárez J , Díaz-de Alba P , Rodríguez Iglesias M , Pascual A . 2020. First identification of blaNDM-1 carbapenemase in blaOXA-94-producing Acinetobacter baumannii ST85 in Spain. Enfermedades Infecc Microbiol Clínica 38:11–15. doi:10.1016/j.eimc.2019.03.008 31060865

[67] Zafer MM , Hussein AFA , Al-Agamy MH , Radwan HH , Hamed SM . 2021. Genomic characterization of extensively drug-resistant NDM-producing Acinetobacter baumannii clinical isolates with the emergence of novel BLA ADC-257. Front Microbiol 12:736982. doi:10.3389/fmicb.2021.736982 34880837 PMC8645854

[68] Jaidane N , Naas T , Oueslati S , Bernabeu S , Boujaafar N , Bouallegue O , Bonnin RA . 2018. Whole-genome sequencing of NDM-1-producing ST85 Acinetobacter baumannii isolates from Tunisia. Int J Antimicrob Agents 52:916–921. doi:10.1016/j.ijantimicag.2018.05.017 29857033

[69] Maamar E , Alonso CA , Ferjani S , Jendoubi A , Hamzaoui Z , Jebri A , Saidani M , Ghedira S , Torres C , Boubaker IB-B . 2018. NDM-1- and OXA-23-producing Acinetobacter baumannii isolated from intensive care unit patients in Tunisia. Int J Antimicrob Agents 52:910–915. doi:10.1016/j.ijantimicag.2018.04.008 29665444

[70] Maciel WG , da Silva KE , Croda J , Cayô R , Ramos AC , de Sales RO , de Almeida de Souza GH , Bampi JVB , Limiere LC , Casagrande JC , Gales AC , Simionatto S . 2018. Clonal spread of carbapenem-resistant Acinetobacter baumannii in a neonatal intensive care unit. J Hosp Infect 98:300–304. doi:10.1016/j.jhin.2017.10.015 29107079

[71] Stratev A , Tanova R , Dimov S , Mitov I , Strateva T . 2020. Clonal spread of carbapenem-resistant Acinetobacter baumannii isolates among Bulgarian critically ill patients undergoing renal replacement therapy (2016–2018). Infect Dis 52:430–433. doi:10.1080/23744235.2020.1725622 32043409

[72] Zhang X , Li F , Awan F , Jiang H , Zeng Z , Lv W . 2021. Molecular epidemiology and clone transmission of carbapenem-resistant Acinetobacter baumannii in ICU rooms. Front Cell Infect Microbiol 11:633817. doi:10.3389/fcimb.2021.633817 33718283 PMC7952536

[73] Huang L , Sun L , Yan Y . 2013. Clonal spread of carbapenem resistant Acinetobacter baumannii ST92 in a Chinese Hospital during a 6-year period. J Microbiol 51:113–117. doi:10.1007/s12275-013-2341-4 23456719

[74] Matsui M , Suzuki M , Suzuki M , Yatsuyanagi J , Watahiki M , Hiraki Y , Kawano F , Tsutsui A , Shibayama K , Suzuki S . 2018. Distribution and molecular characterization of Acinetobacter baumannii International clone II lineage in Japan. Antimicrob Agents Chemother 62:e02190-17. doi:10.1128/AAC.02190-17 29203489 PMC5786803

[75] Zarrilli R , Pournaras S , Giannouli M , Tsakris A . 2013. Global evolution of multidrug-resistant Acinetobacter baumannii clonal lineages. Int J Antimicrob Agents 41:11–19. doi:10.1016/j.ijantimicag.2012.09.008 23127486

[76] Jun SH , Lee DE , Hwang HR , Kim N , Kim HJ , Lee YC , Kim YK , Lee JC . 2021. Clonal change of carbapenem-resistant Acinetobacter baumannii isolates in a Korean hospital. Infect Genet Evol 93:104935. doi:10.1016/j.meegid.2021.104935 34029723

[77] Sahl JW , Del Franco M , Pournaras S , Colman RE , Karah N , Dijkshoorn L , Zarrilli R . 2015. Phylogenetic and genomic diversity in isolates from the globally distributed Acinetobacter baumannii ST25 lineage. Sci Rep 5:15188. doi:10.1038/srep15188 26462752 PMC4604477

[78] Bontron S , Nordmann P , Poirel L . 2016. Transposition of Tn125 Encoding the NDM-1 carbapenemase in Acinetobacter baumannii. Antimicrob Agents Chemother 60:7245–7251. doi:10.1128/AAC.01755-16 27671058 PMC5119004

[79] Li L , Dechesne A , He Z , Madsen JS , Nesme J , Sørensen SJ , Smets BF . 2018. Estimating the transfer range of plasmids encoding antimicrobial resistance in a wastewater treatment plant microbial community. Environ Sci Technol Lett 5:260–265. doi:10.1021/acs.estlett.8b00105

