# Supplementary material for: NDM-1- and OXA-23-producing Acinetobacter baumannii in wastewater of a Nigerian hospital
Source: Microbiol Spectr. 2023 Oct 5;11(6):e02381-23. doi: 10.1128/spectrum.02381-23 (PMC10714947; doi:10.1128/spectrum.02381-23)
Supplement: Supplemental figures — Fig. S1 and S2. [file spectrum.02381-23-s0001.docx]

Supplementary Figures


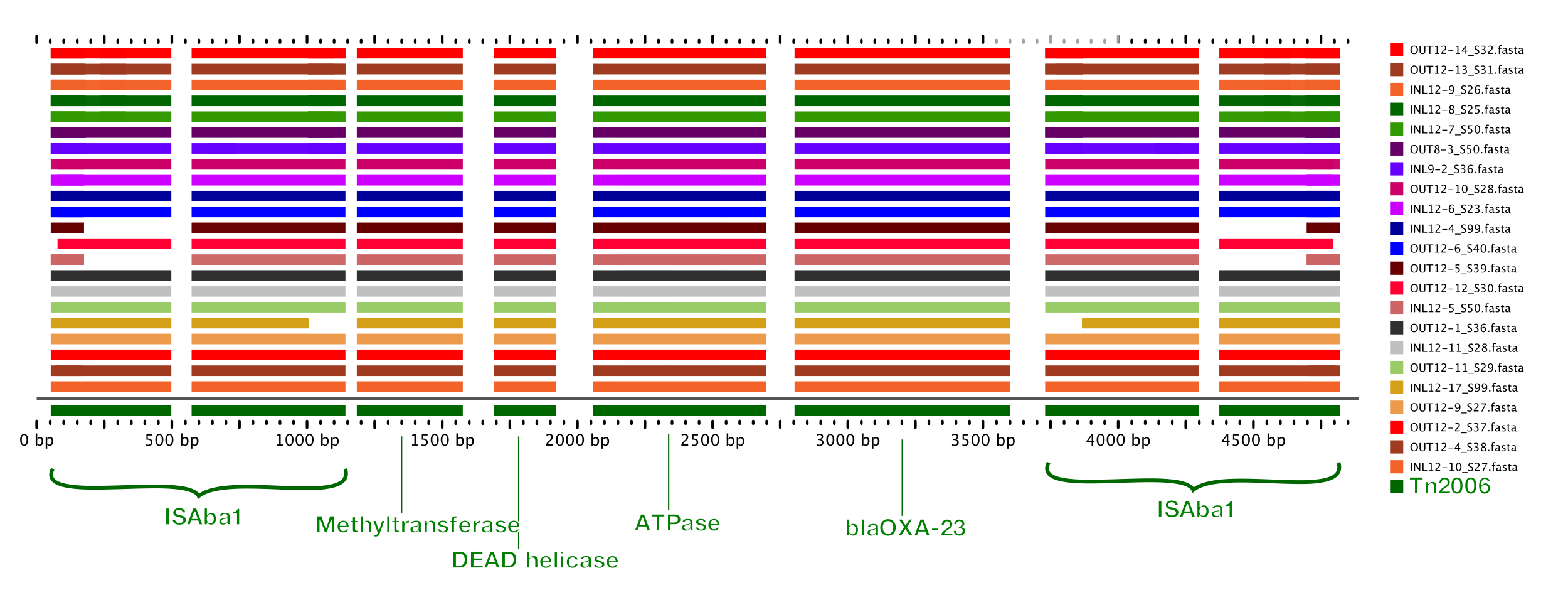


Figure S1: Genome assemblies of the 22 *bla*_OXA-23_-positive *A. baumannii* isolates mapped to transposon Tn2006.


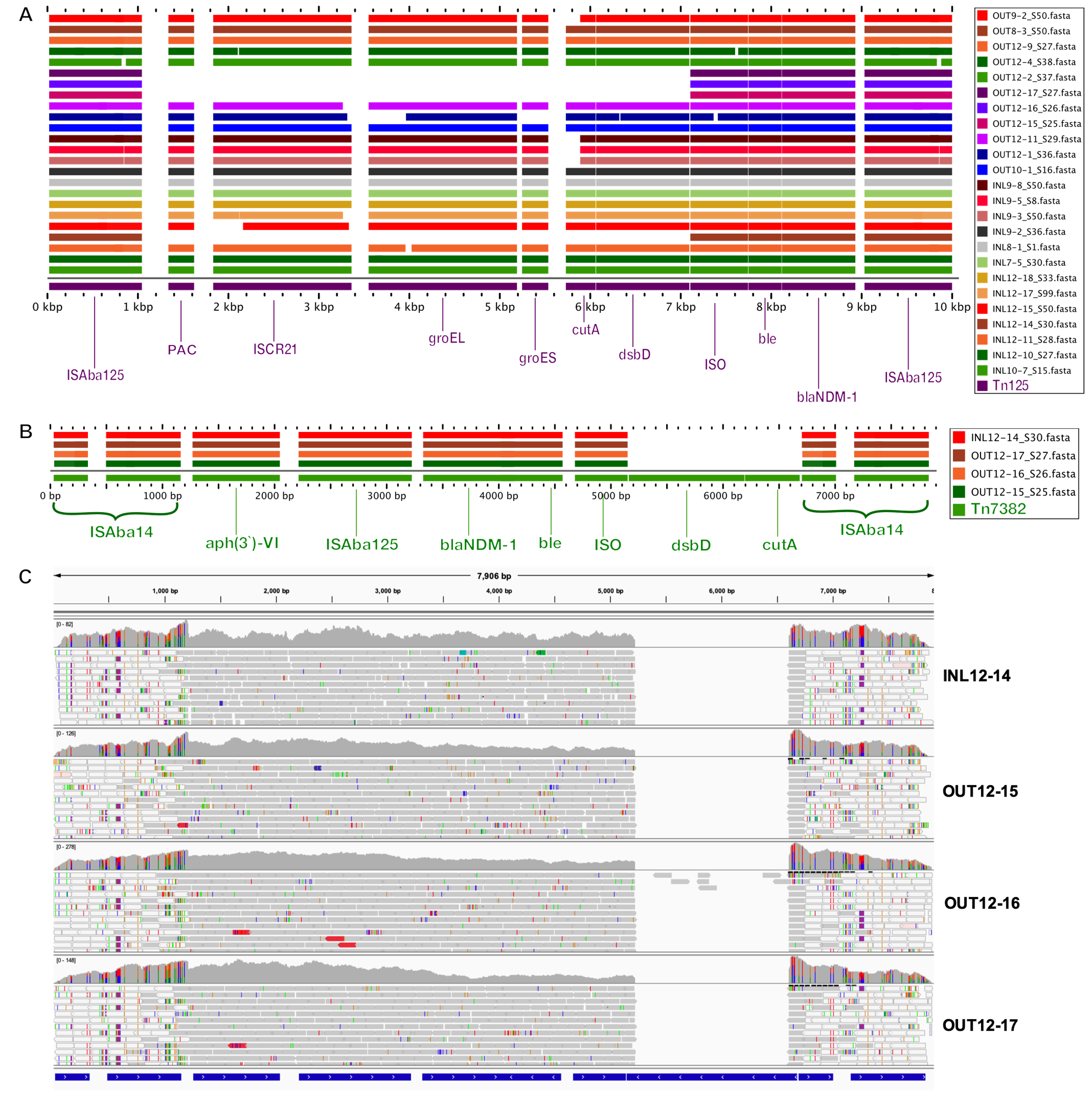


Figure S2: (A) Genome assemblies of the 24 *bla*_NDM-1_-positive *A. baumannii* isolates mapped to transposon Tn*125*. All but the four ST472 isolates shown to carry Tn*125* or Tn*125*-like transposons (B) Assemblies of the four ST472 isolates mapped to transposon Tn*7382* and showing a Tn7382-like structure with missing *dsbD* and *cutA* genes (C) Sequence reads of the four ST472 isolates mapped to transposon Tn*7382*
